# Age-Based Differences in the Genetic Determinants of Glycemic Control: A Case of *FOXO3* Variations

**DOI:** 10.1371/journal.pone.0126696

**Published:** 2015-05-20

**Authors:** Liang Sun, Caiyou Hu, Yu Qian, Chenguang Zheng, Qinghua Liang, Zeping Lv, Zezhi Huang, Keyan Qi, Jin Huang, Qin Zhou, Ze Yang

**Affiliations:** 1 The key Laboratory of Geriatrics, Beijing Hospital and Beijing Institute of Geriatrics, Ministry of Health, Beijing, China; 2 Department of Neurology, Jiangbin Hospital, Nanning, Guangxi, China; 3 Key Laboratory of Biorheological Science and Technology, Ministry of Education, Bioengineering College, Chongqing University, Chongqing, China; 4 Department of Cardiothoracic Surgery, Guangxi Maternal and Child Health Hospital, Nanning, Guangxi, China; 5 Office of longevity cultural, People’s government of Yongfu County, Yongfu, Guangxi, China; 6 Lab of Genetics and Metabolism, Beijing Obstetrics and Gynecology Hospital, Beijing, China; 7 Department of obstetrics, Beijing Shunyi Airport Hospital, Beijing, China; Virgen Macarena University Hospital, School of Medicine, University of Seville, SPAIN

## Abstract

**Background:**

Glucose homeostasis is a trait of healthy ageing and is crucial to the elderly, but less consideration has been given to the age composition in most studies involving genetics and hyperglycemia.

**Methods:**

Seven variants in *FOXO3* were genotyped in three cohorts (n = 2037; LLI, MI_S and MI_N; mean age: 92.5±3.6, 45.9±8.2 and 46.8±10.3, respectively) to compare the contribution of *FOXO3* to fasting hyperglycemia (FH) between long-lived individuals (LLI, aged over 90 years) and middle-aged subjects (aged from 35–65 years).

**Results:**

A different genetic predisposition of *FOXO3* alleles to FH was observed between LLI and both of two middle-aged cohorts. In the LLI cohort, the longevity beneficial alleles of three variants with the haplotype “AGGC” in block 1 were significantly protective to FH, fasting glucose, hemoglobin A_1C_ and HOMA-IR. Notably, combining multifactor dimensionality reduction and logistic regression, we identified a significant 3-factor interaction model (rs2802288, rs2802292 and moderate physical activity) associated with lower FH risk. However, not all of the findings were replicated in the two middle-aged cohorts.

**Conclusion:**

Our data provides a novel insight into the inconsistent genetic determinants between middle-aged and LLI subjects. *FOXO3* might act as a shared genetic predisposition to hyperglycemia and lifespan.

## Introduction

Glucose homeostasis is a trait of healthy aging and is crucial to the elderly. Fasting hyperglycemia (FH) is the most convenient and practical predictor of overall glycemic control monitoring throughout the day [1]. Suboptimal control of fasting hyperglycemia could increase the risk of cardiovascular complications in elderly subjects [2, 3]. In the United States, fasting hyperglycemia causes excess morbidity and increased health-care costs and affects approximately one in five (18.7%) persons aged >65 years; further, as the population ages, the impact of hyperglycemia intensifies [4]. Indeed, fasting hyperglycemia is also considered an independent predictor of mortality [5].

Only a few of long-lived individuals (LLI) have been involved in most studies involving the genetics of ageing-related disorders. Epidemiological evidence suggests that different genetic risk factors are involved throughout the course of human life [6]. The American Diabetes Association (ADA) and the European Association for the Study of Diabetes (EASD) have suggested that the management of hyperglycemia must be individualized [7]. Therefore, it is necessary to re-characterize the risk factors of fasting hyperglycemia that are specific to elderly subjects.

Glucose homeostasis is directly caused by the dysfunction of the insulin/IGF-1 signaling (IIS) pathway [8]. Forkhead box class O3 (FOXO3), as the *daf-16* human homologue, is the main target of the IIS pathway [9]. *FOXO3* has been recognized as a “master” gene in Europeans [10–12], East Asians [13] and Ashkenazi Jews [14], although its potential mechanism is unclear. It is worth noting that the *Cis*-element of the *FOXO3* binding site has been identified in the promoters of the gluconeogenesis genes *G6Pase* and *PEPCK* [15], implicating it in fasting glucose control. However, no supportive evidence of *FOXO3* variants has been reported to confer the risk for fasting hyperglycemia or type 2 diabetes (T2D) in middle-aged subjects of Indian origin [16]. Moreover, potential interactions between *FOXO3* variants and other longevity-related factors, such as APOE*e4 [17], physical activity (PA) [18] and lipid levels [19], have been even less well studied.

Here, we performed a comprehensive analysis of *FOXO3* and fasting hyperglycemia in three cohorts (n = 2037). The tagging single nucleotide polymorphism (tSNP) approach was used to compare the contribution of *FOXO3* to risk of fasting hyperglycemia between LLI and middle-aged (MI_S and MI_N) subjects. The interactions between *FOXO3* variants and other longevity-related factors were further addressed.

## Materials and Methods

### Study population

The project is a cross-sectional study within the framework of the “Longevity and Health of Aging Population in Guangxi China (LHAPGC)” and “Beijing Type 2 Diabetes Studies (BTDS)” [20, 21]. A total of 2,037 Han Chinese are included, consisting of three panels: 506 LLI (centenarians and nonagenarians) (mean age: 92.5±3.6 years) and 1,531 middle-aged subjects (830 southern Chinese, MI_S, mean age: 45.9±8.2 years; 701 northern Chinese, MI_N, mean age: 46.8±10.3 years). According to the 1999 World Health Organization (WHO) diagnostic criteria [22], the subjects were defined as FH (Impaired Fasting Glycaemia/ T2D) or normal controls (fasting plasma glucose < = 6.0 mmol/l). Subjects being treated with oral hypoglycemic agents (OHA) were excluded. The Ethics Committee of Beijing Hospital, Ministry of Health approved the study protocol. All participants were informed and provided informed consent in writing.

### Data collection and laboratory measurements

The peripheral blood was collected for DNA extraction and laboratory biochemistry testing according to previously described methods [23]. Genomic DNA was isolated using a standardized salting-out procedure. Clinical biomarkers including fasting glucose (FPG), hemoglobin A_1C_ (HBA_1C_), insulin (FINS), total cholesterol (TC), triglyceride (TG), HDL-cholesterol, LDL-cholesterol and APOE*e4 status [24] were assessed. The insulin resistance status was assessed through a homeostasis model assessment of insulin resistance (HOMA-IR), calculated as FPG (mM)×FINS (mIU/L) /22.5 [25].

Trained and qualified personnel conducted the households and home visits. Potential confounding factors included gender, current drinking (yes, no), current smoking (yes, no), physical activity (PA) (no exercise, light, moderate, heavy), education and family history of chronic diseases (coronary heart disease, stroke, hypertension or diabetes) were assessed using a self-report questionnaire.

### SNP selection and genotyping

Based on the CHB population (Han Chinese residents in Beijing) data, we selected seven tSNP covering 120.9 kb on chromosome 6 by HapMap online tools according to: (1) minor allele frequency (MAF) >0.05; (2) an r^2^ threshold of 0.8 and a log of odds (LOD) threshold for multi-marker testing of 3.0; (3) a minimum distance between tags of 100 bp; (4) SNPs for which an association with longevity has been reported; and (5) both pairwise tagging and aggressive multi-marker tagging (use 2-marker haplotypes) strategies were conducted to increase the efficiency of tagging.

The tSNPs were genotyped by a small amplicon-based high resolution melting (HRM) assay with LightScanner (Idaho Technology, Alameda, CA, USA) (S1 Table) [26]. To ensure the reliability of the results, duplicate samples and sequencing verified genotyped samples were included as quality controls.

### Statistical analysis

SPSS software (version 20.0) was used for analysis. Both of clinical fasting hyperglycemia diagnosis and tertiles of levels of FPG, HbA_1c_ and HOMA-IR were used for the comparisons. One-way ANOVA was used to compare continuous parameters among three groups followed by Tukey’s post-hoc tests. Logistic regression was performed to compare minor allele frequency (MAF) after adjusting for confounding factors (*e*.*g*., gender, drinking, smoking, PA). An allele contrast model was used, unless specified. Haploview (version 4.2) was used to estimate the Hardy-Weinberg equilibrium, linkage disequilibrium (LD) and common haplotype (>3%) among genotyped variants. A two-tailed *P*<0.05 was considered statistically significant. Power calculations were performed with an interactive program, Power and Sample size Calculation (version 3.0) [27].To assess interactions between *FOXO3* genotype and other longevity-related factors (physical activity/ *APOE4*/ lipid levels), respective multiplicative interaction terms were included in regression models. An additive model was used to code SNPs for the ‘risk’ allele, the allele that increased the probability of being a case. Accordingly, ‘0’ indicated the subject was homozygous for the non-risk allele; the heterozygote was coded ‘1’, and those who were homozygous for the risk allele were coded ‘2’. Multifactor Dimensionality Reduction (MDR, version 1.2.2) was also used to confirm the interaction (epistasis) between genetic and other longevity-related factors (permutation n = 8000) to determine the statistical significance of the best models. The testing balanced accuracy (TBA) and cross-validation consistency (CVC) were estimated. These data were also analyzed using an extension of the MDR algorithm that includes adjustment for covariates, the Generalized Multifactor Dimensionality Reduction (GMDR, V0.7) software package.

## Results

### Participant Characteristics for the Cohorts

Characteristics of three panels of involved participants (506 LLI, 830 MI_S and 701 MI_N) are presented in Table 1. Generally, the prevalence of fasting hyperglycemia is higher in middle-aged subjects than it is in long-lived individuals. In addition, subjects in the long-lived individuals cohort have lower levels of FPG, HbA_1c_, HOMA-IR, BMI, TC, TG, and proportion of APOE*e4 than middle-aged subjects, except for higher blood pressure and LDL-cholesterol.

**Table 1:** Demographic and metabolic characteristics of the study samples.

	LLI	MI_S	MI_N	*P*_*post-hoc1*_ ^*a*^	*P*_*post-hoc2*_ ^*b*^
AGE (years)	92.5±3.6	45.9±8.2	46.8±10.3	<0.001	<0.001
Samples (n)	506	830	701	NA	NA
Gender (male %) ^d^	26.5	66.3	55.9	<0.001	<0.001
FH (%)^d^	42.0	48.1	44.7	0.028	0.341
FPG (mmol/L)	5.7±1.4	6.3±1.4	5.9±0.9	0.021	0.049
HbA_1c_ (%)	5.9±1.8	6.1±2.2	6.0±2.6	0.033	0.027
HOMA-IR^c^	2.2±0.4	2.6±1.1	2.4±0.7	0.014	0.018
BMI (kg/m^2^)	18.6±3.7	23.6±3.2	23.5±4.5	<0.001	<0.001
SBP (mmHg)	149.1±23.0	124.1±16.2	118.1±16.1	<0.001	<0.001
DBP (mmHg)	79.9±12.2	76.1±10.6	74.2±10.6	0.017	<0.001
TC (mmol/L)	4.8±1.1	5.1±0.9	4.9±0.3	0.029	0.048
TG (mmol/L)	1.3±0.9	2.6±1.3	2.4±1.1	<0.001	<0.001
HDL-cholesterol (mmol/L)	1.3±0.3	1.2±0.2	1.2±0.2	0.347	0.281
LDL-cholesterol (mmol/L)	2.9±0.9	2.7±0.8	2.4±0.5	0.033	0.026
APOE*e4 carriers (%)^d^	14.6	18.9	21.3	0.044	0.003

Data are shown as the mean±SD or n; FH: fasting hyperglycemia; LLI: long-lived individuals; MI_S: middle-aged controls (southern); MI_N: middle-aged (northern); HbA_1c_: hemoglobin A_1c_; HOMA-IR: homeostasis model assessment of insulin resistance; BMI: body mass index; SBP: systolic blood pressure; DBP: diastolic blood pressure; TC: total cholesterol; TG: triglyceride; HDL: high density lipoprotein; LDL: low density lipoprotein; NA: not available.

^a^ *p* values of post-hoc pair-wise comparisons of the parameters between LLI and MI_S.

^b^ *p* values of post-hoc pair-wise comparisons of the parameters between LLI and MI_N.

^c^ after log-transformed to normalize skewed distribution.

^d^ comparison of categorical variable based on Chi-square test.

### FOXO3 Genotype and Haplotype Analysis

Given subject number in each group and an average allelic relative risk of 0.67, we had 82.2%, 96.3% and 92.9% power to detect a difference of a significance level of 5% corresponding to LLI, MI_S and MI_N (assumed MAF = 0.35).

**Fig 1:**
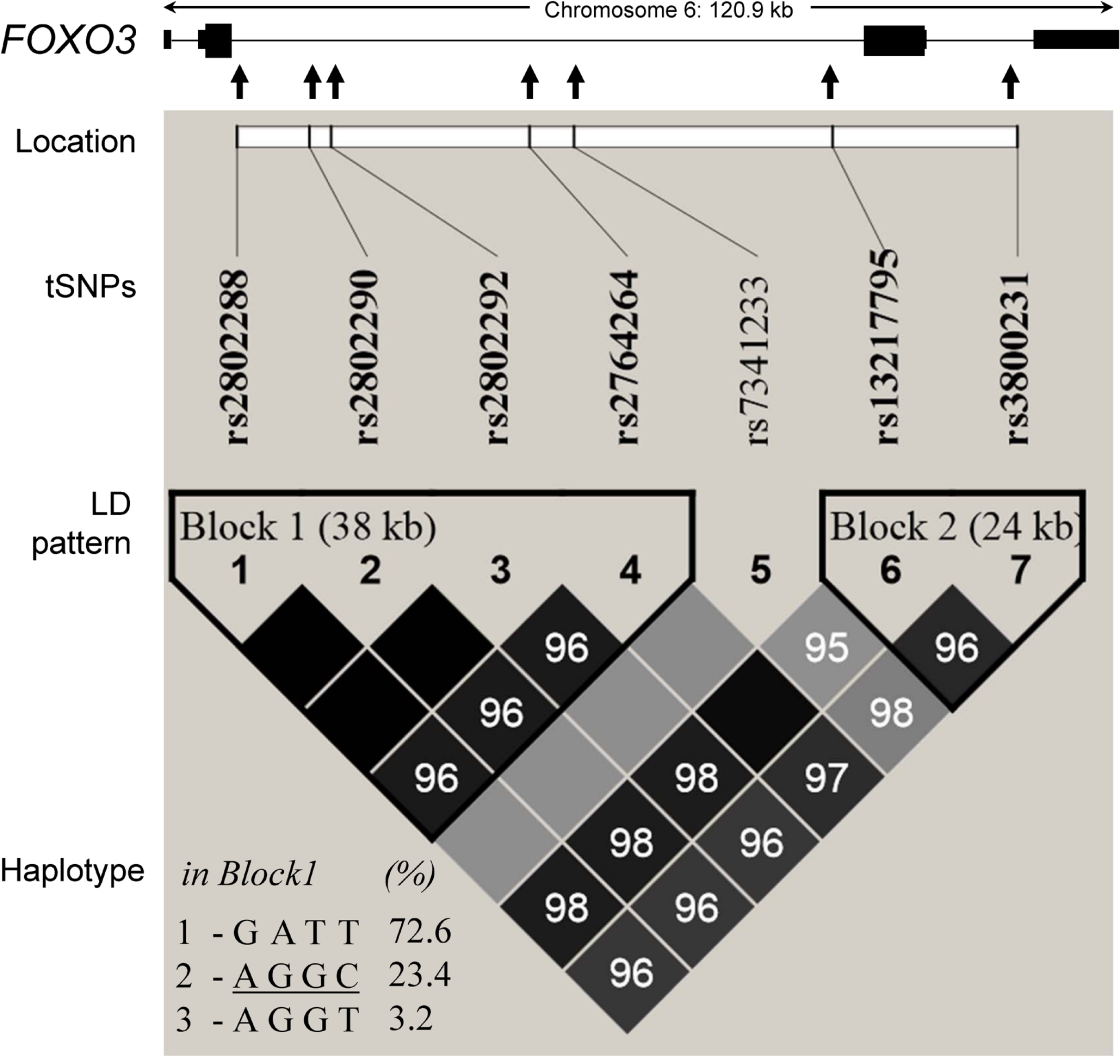
Graphical representation of the polymorphisms and LD structure across *FOXO3* in the subjects of this study. The *FOXO3* locus consists of four exons (thin dark-shaded boxes represent UTRs, and thick dark-shaded boxes represent coding exons). The thin lines represent introns. The relative locations of seven variants are listed. Two linkage disequilibrium (LD) blocks are defined. The measures of LD (D’) among paired of variants are shown graphically by color (r^2^). A white box represents very low LD, and a dark box represents very high LD. The constitutions and frequencies of three common haplotypes in block 1 are listed.

According to the extent of LD in *FOXO3* using the HapMap CHB data, seven tSNPs were selected and genotyped that could define most of the variants (S2 Table). All tSNPs were in Hardy-Weinberg equilibrium in non-FH controls of all three cohorts (Table 2). Two haplotype blocks were defined and separated by rs7341233 (Fig 1). The block 1 consisted of the first four variants, which defined three common haplotypes and described 99.2% of haplotype diversity. In both middle-aged panels (MI_S and MI_N), all of the MAFs were similar between FH and non-FH subjects. However, three variants (rs2802288*A, rs2802290*G and rs2802292*G) and haplotype 2 “AGGC” in Block 1(compared with all other haplotypes combined) were associated with decreased risk of fasting hyperglycemia only in long-lived individuals (Tables 2 and 3). Consistently, the FH- protective alleles/ haplotype were also benefit for longevity in general, except for rs2802290 (T/G) (S3 Table).

**Table 2:** Minor allele frequencies of the *FOXO3* tSNPs in three cohorts.

Variants in *FOXO3* ^a^	Position^b^	LLI (n = 506)		MI_S (n = 830)		MI_N (n = 701)	
		MAF	*P* ^*c*^	MAF	*P* ^*c*^	MAF	*P* ^*c*^
rs2802288 (G/A)	109002908	0.33/0.41	0.014	0.31/0.33	0.310	0.29/0.32	0.170
rs2802290 (A/G)	109012373	0.30/0.36	0.040	0.31/0.34	0.151	0.29/0.33	0.097
rs2802292 (T/G)	109015211	0.31/0.38	0.019	0.29/0.33	0.077	0.30/0.32	0.267
rs2764264 (T/C)	109041154	0.33/0.39	0.053	0.30/0.33	0.173	0.29/0.31	0.293
rs7341233 (T/C)	109046973	0.17/0.18	0.717	0.18/0.19	0.646	0.18/0.19	0.657
rs13217795 (T/C)	109080791	0.30/0.35	0.111	0.27/0.30	0.335	0.25/0.27	0.336
rs3800231 (G/A)	109104959	0.33/0.36	0.281	0.29/0.31	0.244	0.28/0.30	0.450

MAF: minor allele frequency; FH: fasting hyperglycemia; LLI: long-lived individuals; MI_S: middle-aged controls (southern); MI_N: middle-aged (northern).

^a^ the minor allele corresponds to the latter base in each description of variants, separated by a “/”.

^b^ Position on Chromosome 6 (GRCh36/hg18).

^c^ Comparisons under allele-contrast model between FH cases and contros.

**Table 3:** Comparisons of effects on risk of FH between LLI and middle-aged subjects.

Comparison	LLI (n = 506)		MI_S (n = 830)		MI_N (n = 701)	
	OR (95%CI)	*P*	OR (95%CI)	*P*	OR (95%CI)	*P*
rs2802288 (G/A)						
Model_cca_	0.72 (0.56–0.94)	0.014	0.90 (0.73–1.10)	0.310	0.85 (0.68–1.07)	0.170
Model_adj_	0.77 (0.50–0.99)	0.019	0.94 (0.77–1.16)	0.420	0.91 (0.72–1.14)	0.372
rs2802290 (A/G)						
Model_cca_	0.76 (0.58–0.99)	0.040	0.86 (0.70–1.06)	0.151	0.83 (0.66–1.04)	0.097
Model_adj_	0.81 (0.62–1.05)	0.128	0.90 (0.74–1.11)	0.214	0.94 (0.75–1.17)	0.301
rs2802292 (T/G)						
Model_cca_	0.73 (0.56–0.95)	0.019	0.83(0.67–1.02)	0.077	0.88 (0.70–1.10)	0.267
Model_adj_	0.67 (0.51–0.87)	0.012	0.92 (0.75–1.13)	0.171	1.04 (0.83–1.30)	0.349
Haplotype2:AGGC ^a^						
Model_perm_	0.67 (0.51–0.87)	0.019	1.09 (0.83–1.42)	0.441	1.26(0.96–1.65)	0.496
Model_adj_	0.74 (0.57–0.95)	0.043	1.12 (0.85–1.47)	0.462	0.97 (0.74–1.28)	0.253

LLI: long-lived individuals; MI_S: middle-aged controls (southern); MI_N: middle-aged (northern); FH: fasting hyperglycemia;Model_cca_, Model_adj_ and Model_perm_ corresponded to allele contrast, adjusted for confounding factors (BMI, age, gender, drinking, current smoking and APOE*e4 status) and permutation corrections for multiple comparisons (n = 8000).

^a^ between Haplotype2 and all other haplotypes combine.

### FOXO3 Variants and Hyperglycemia related Clinical Parameters Analysis

**Fig 2:**
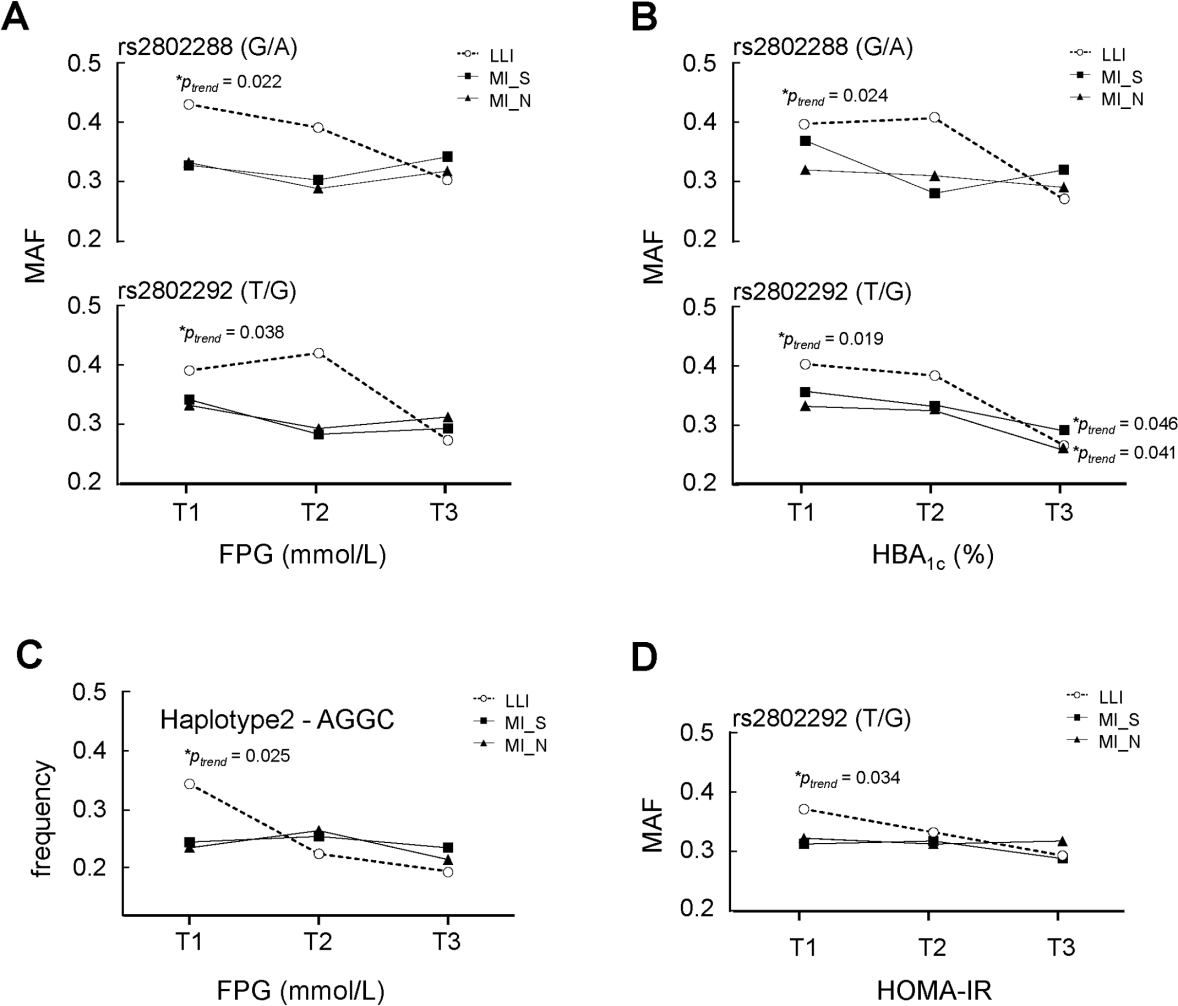
Comparisons of alleles and haplotypes in *FOXO3* and tertiles of hyperglycemia related parameters in each cohort. MAF: minor allele frequency; T1,T2 and T3 represents the lowest, middle and highest tertile, respectively. *p-values* were obtained from logistic regression analysis adjusted for age and gender. Only in LLI, was a significant contribution of *FOXO3* found. Both frequencies of rs2802288*A and rs2802292*G were reduced when A) fasting plasma glucose and B) HbA_1c_ increased. C) The frequency of Haplotype 2 “AGGC” in block 1 decreased when fasting plasma glucose increased. D) The frequency of rs2802292*G decreased when HOMA-IR increased.

As expected, only in long-lived individuals, subjects in the top tertile of both FPG and HbA_1c_ experienced lower MAFs of rs2802288 and rs2802292 than did patients of the other two tertiles (*p*<0.05). Marginal differences were found with respect to rs2802292*G across the tertiles of HOMA-IR. Carriers with haplotype 2 “AGGC” had a trend for lower FPG levels as well (Fig 2). However, except for a slight difference between rs2802292*G and HbA_1c_ in both middle-aged cohorts, no other relationship was observed among the lipid profiles, the risk for T2DM, obesity and other cardiovascular diseases in all three cohorts for any *FOXO3* variant and haplotype (S4 Table).

### Gene-Gene and Gene-Environment Interactions

The interaction between *FOXO3*, APOE*e4, and environmental factors (gender, drinking, smoking, PA, education, and family history of chronic diseases) was further analyzed. Only rs2802288*A and rs2802292*G in block 1 were included because of the strong LD pattern. The 3-factor model comprised of rs2802288*A, rs2802292*G and moderate PA emerged as the best fasting hyperglycemia predicting model with the highest level of statistical significance (MDR: TBA = 0.571, CVC = 9/10, *p* = 0.007; GMDR adjusted for age, BMI and alcohol drinking: TBA = 0.542, CVC = 9/10, *p* = 0.009). The nonparametric MDR analysis indicated that the 3-factor model was the best model but did not indicate whether there was a synergistic relationship. Therefore, we applied a parametric logistic regression analysis and observed an interaction (among rs2802288*A, rs2802292*G and moderate PA) that was inversely associated with risk of fasting hyperglycemia (*p* = 0.003, OR = 0.589, 95%CI = 0.418–0.831) only in long-lived individuals. The analysis of MDR and logistic regression both suggested the *FOXO3*-PA interaction might have a bigger effect than if considered independently. However, no correlation was observed in either middle-aged panel.

## Discussion

Although age is important in the pathogenesis of hyperglycemia, most previous studies have included only low numbers of long-lived individuals. In the present study, for the first time, we report the different genetic impact of *FOXO3* on fasting hyperglycemia as an intermediated trait of healthy aging in long-lived individuals compared with middle-aged subjects (MI_S, MI_N).

In long-lived individuals, both single allele and haplotype analysis suggested a genetic association between *FOXO3* block 1 and fasting hyperglycemia. Moreover, a 3-factor interaction between rs2802288*A and rs2802292*G in *FOXO3* and moderate PA was identified by nonparametric MDR and logistic regression. The variants were also associated with improved glycemic traits with lower FPG and HbA_1c_ levels (rs2802288*A, rs2802292*G, and haplotype2 “AGGC”) and HOMA-IR (rs2802292*G) in long-lived individuals. In contrast, all the findings did not exist in both middle-aged panels (MI_S, MI_N).

In a small Danish twin cohort, the rs2802292*G was found to enhance insulin sensitivity with increased skeletal muscle-*FOXO3* expression, although it has failed to be replicated in a middle-aged Inter99 cohort (aged 46±8) [28]. In this study, we also observed that rs2802292*G was associated with decreased risk of fasting hyperglycemia, lower levels of hyperglycemia (FPG and HBA1c), and improved HOMA-IR only in long-lived individuals. In a middle-aged South Indian Dravidian population, Nair A K *et al*. failed to find contribution of *FOXO3* to T2DM; the carriers with rs2802288*A also seemed to have lower FPG and BMI, indicating improved insulin resistance [16]. Meanwhile, rs2802288 was in complete LD with rs2802292. Considering the strong LD in Block 1 and clues of individual variants in this study, we suspected that rs2802292*G might act as the major contributor to fasting hyperglycemia.

We found an interaction between *FOXO3* variants and moderate physical activity on fasting hyperglycemia in long-lived individuals. Since moderate physical activity could improve the insulin resistance and further uptake of glucose in skeletal muscles, it is reasonable for subjects who are used to moderate physical activity possess lower fasting plasma glucose and better insulin sensitivity. Evidence in animal models also indicated that *FOXO3* phosphorylation plays crucial role in the metabolism improvement induced by exercise [29, 30]. In older subjects, there might be a “beneficial cycle” that subjects without chronic health conditions could do more moderate physical exercise to be healthier, that might partly be the reason for the interaction identified in long-lived individuals.

*FOXO3* variants might acts in “modest effector model” in human and affect sub-clinical traits. As a housekeeping gene, *FOXO3* is evolutionarily conserved [28], so a missense mutation might lead to severe phenotypes and premature death. Consistently, all the existing studies only identified the noncoding variants in *FOXO3* [31]. This could partly account for why we failed to find the genetic contribution of *FOXO3* to T2DM, obesity and metabolic syndrome, possibly because of the minor effect of *FOXO3* variants which is insufficient for the threshold of a disease. It might play a role in multiple sub-clinical traits instead of furthering a disease itself. It would be worth running the same analysis in non-T2D cohorts to minimize bias.

**Fig 3:**
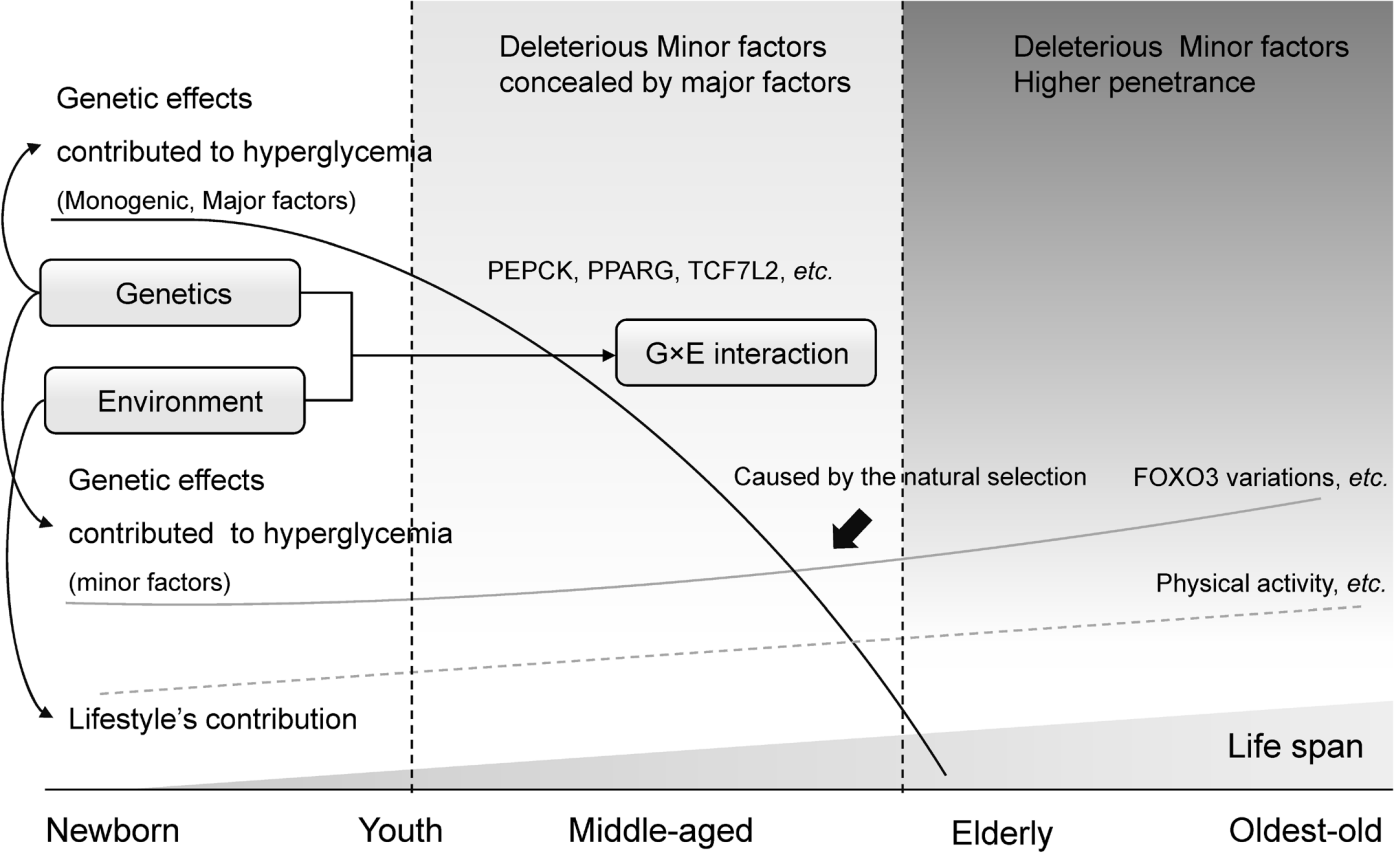
Schematic of the potential hypothesis for an inconsistence in genetic and environmental determinants across human lifespan. SNPs having more severe effects on glucose homeostasis are likely to have resulted in earlier mortality, removing them/their effects from the long-lived cohort, and that this allows them to easier detect other variants having more modest effects.

*FOXO3* alleles have been associated with human longevity across ethnicities [10–14]. Interestingly, the genetic determinants identified in this study were largely those associated with human longevity (S5 Table). In theory, most of the aging-related intermediate traits and diseases derive from the gradual accumulation of genetic and environmental factors across the whole lifespan. This present study suggests a very good case for an inconsistent effect of genetic determinants on ageing-related mediated traits across different ages (Fig 3). Variants having more severe effects on homeostasis are likely to have resulted in earlier mortality, removing them/their effects from the long-lived cohort, and that this allows them to easier detect other variants having more modest effects. For hyperglycemia, the effect of modest variants in *FOXO3* might be concealed by other major variants (*e*.*g*., *PEPCK*, *PPARG* and *TCF7L2*) in most studies based on middle-aged subjects. However, it might be different when subject enter their eighties, or become even older, for the selection by death of major deleterious factors, whereas the long-lived individuals subjects could survive. Accordingly, it is unreasonable to apply the findings from middle-aged subjects to the long-lived individuals directly. Therefore, when long-lived individuals are investigated, the pattern of genetics should be re-considered, similar to the distinct pattern present in children.

The first strength of this study is the connection of shared alleles between lifespan and intermediate phenotypes in human, which effects differently in long-lived individuals and middle-aged subjects. Second, environmental factors (such as drinking, smoking, PA) were considered, including the potential interaction with *FOXO3* variants estimated using the nonparametric MDR method combined with multivariable-adjusted logistic regression. There are limitations in most retrospective association studies, including our current study. First, the insulin-related clinical parameters were incomplete. Because of the relative frail status of long-lived individuals with an increased risk of adverse events of hyperglycemic clamp and oral glucose tolerance test, only FPG, FINS, HOMA-IR and HbA_1c_ were assessed. Second, the clinical relevance including the mortality, such as the Leiden 85-plus study, was not addressed comprehensively, which will be emphasized in our next global long-term follow-up work. Third, further next-generation sequencing-based exome scanning is needed to pinpoint the location of causal variant. However, this cross-sectional data would help direct the next follow-up visits.

In summary, our data provide a novel insight into the inconsistent genetic determinants between mid-life and long-lived individuals. The protective contribution of *FOXO3* and PA to fasting hyperglycemia only existed in our long-lived individuals, suggesting that *FOXO3* might be a shared genetic predisposition between hyperglycemia and lifespan.

## Supporting Information

S1 Table Primers for genotyping of *FOXO3* polymorphisms based on High-Resolution Melting Assay. H_calibrator: high temperature calibrator (3’-C3-blocked); L_ calibrator: low temperature calibrator (3’-C3-blocked); NA: not available.
(DOCX)

S2 Table Variants in *FOXO3* captured by the tSNP design based on the HapMap database. Seven tSNP covering the 120.9kb on chromosome 6 by HapMap online tools based on the CHB population (Han Chinese residents in Beijing) data. * criteria for inclusion: (1) minor allele frequency (MAF) >0.05; (2) r2 threshold of 0.8 and a log of odds (LOD) threshold for multi-marker testing of 3.0; 3) a minimum distance between tags of 100 bp; (4) SNPs for which an association with longevity has been reported were forced included; (5) both of pairwise tagging and aggressive multi-marker tagging (use 2-marker haplotypes) strategies were conducted to increase the efficiency of tagging.
(DOCX)

S3 Table The association of fasting hyperglycemia-protective alleles/ haplotype in *FOXO3* with longevity. Comparisons between LLI and MI_S group, the OR for rs2802288 (G/A), rs2802290 (A/G) and rs2802292 (T/G) were all based on allele-contrast model.
(DOCX)

S4 Table Relationship between effect alleles in *FOXO3* and lipids, T2DM, Obesity and CVD status. TC: total cholesterol; TG: triglyceride; T2DM: type 2 diabetes; CVD: cardiovascular disease; NA: not available for missing data. ^a^ for continuous variables (TC,TG), divided by lowest, middle and highest tertile and calculated by trend chi-square test; for categorical variables (T2DM, Obesity and CVD), calculated by case-control chi-square test. ^b^ according to whether BMI ≥28 kg/m^2^. ^c^ self-reported CVD status.
(DOCX)

S5 Table Publications about the included tSNPs in *FOXO3*. NA: not available.
(DOCX)
