# Supplementary material for: Age-Based Differences in the Genetic Determinants of Glycemic Control: A Case of FOXO3 Variations
Source: PLoS One. 2015 May 20;10(5):e0126696. doi: 10.1371/journal.pone.0126696 (PMC4439071; doi:10.1371/journal.pone.0126696)
Supplement: S1 Table — H_calibrator: high temperature calibrator (3’-C3-blocked); L_ calibrator: low temperature calibrator (3’-C3-blocked); NA: not available. (DOCX) [file pone.0126696.s001.docx]

| SNP (rsID) | Amplicon length (bp) | Primer sequence (5’ to 3’) | |
| --- | --- | --- | --- |
| rs2802288 | 63 | Forward | GGGAGGACTGTGTGGCT |
|  |  | Reverse | TTAAAAGTCCACAGTGGCACT |
| rs2802290 | 58 | Forward | ACAGACCCTGCATGATGGATT |
|  |  | Reverse | CGAAAGGATGGACAACTCCC |
| rs2802292 | 89 | Forward | CTCTACCAGGGTCTCTGTTG |
|  |  | Reverse | TCCCTAGAGAGCAGCAGGA |
| rs2764264 | 52 | Forward | CAGGGTAATGGTGGTCTTATA |
|  |  | Reverse | AGCAGAACAGGGAACACTT |
| rs7341233 | 62 | Forward | CCTATACTGCCTGTTGTCCAA |
|  |  | Reverse | CTAGAAAACCAGGCAAAACAC |
| rs13217795 | 58 | Forward | GCCAACTGAATTACCAAGTAA |
|  |  | Reverse | GGCTCTCCACCTGTCAT |
| rs3800231 | 58 | Forward | ATTTGTCCTCTGCAAGAGTTG |
|  |  | Reverse | GGCTGAATTGGTAGAGGATT |
| H_calibrator | NA | Forward | GCGCGGCCGGCACTGACCCGAGACTCTGAGCGGCTGCTGGAGGTGCGGAAGCGGAGGGGCGGG |
|  |  | Reverse | CCCGCCCCTCCGCTTCCGCACCTCCAGCAGCCGCTCAGAGTCTCGGGTCAGTGCCGGCCGCGC |
| L_ calibrator | NA | Forward | TTAAATTATAAAATATTTATAATATTAATTATATATATATAAATATAATA |
|  |  | Reverse | TATTATATTTATATATATATAATTAATATTATAAATATTTTATAATTTAA |
