# Supplementary material for: Age-Based Differences in the Genetic Determinants of Glycemic Control: A Case of FOXO3 Variations
Source: PLoS One. 2015 May 20;10(5):e0126696. doi: 10.1371/journal.pone.0126696 (PMC4439071; doi:10.1371/journal.pone.0126696)
Supplement: S2 Table — Seven tSNP covering the 120.9kb on chromosome 6 by HapMap online tools based on the CHB population (Han Chinese residents in Beijing) data. * criteria for inclusion: (1) minor allele frequency (MAF) >0.05; (2) r2 threshold of 0.8 and a log of odds (LOD) threshold for multi-marker testing of 3.0; 3) a minimum distance between tags of 100 bp; (4) SNPs for which an association with longevity has been reported were forced included; (5) both of pairwise tagging and aggressive multi-marker tagging (use 2-marker haplotypes) strategies were conducted to increase the efficiency of tagging. (DOCX) [file pone.0126696.s002.docx]

| Variants in *FOXO3* ^*^ | Alleles Captured |
| --- | --- |
| rs2802288 | rs2802288 |
| rs2802290 | rs2802290 |
| rs2802292 | rs2764261, rs768023, rs2490272, rs2802292, rs2253310 |
| rs2764264 | rs2764264 |
| rs7341233 | rs12203787, rs7341233, rs17598747, rs7772662, rs768024, rs12200646, rs12154031, rs7746906, rs12202049, rs3800226, rs12203834, rs17069665, rs12202209, rs12202234, rs12197634, rs3778586, rs12207868, rs12196996, rs11153120, rs17310529, rs12209092, rs12212067 |
| rs13217795 | rs13217795 |
| rs3800231 | rs3800231 |
| rs13217795T* rs2802290A | rs9400239, rs4946932, rs2022464, rs2153960, rs10457180, rs9398171 |
