# Supplementary material for: Age-Based Differences in the Genetic Determinants of Glycemic Control: A Case of FOXO3 Variations
Source: PLoS One. 2015 May 20;10(5):e0126696. doi: 10.1371/journal.pone.0126696 (PMC4439071; doi:10.1371/journal.pone.0126696)
Supplement: S3 Table — Comparisons between LLI and MI_S group, the OR for rs2802288 (G/A), rs2802290 (A/G) and rs2802292 (T/G) were all based on allele-contrast model. (DOCX) [file pone.0126696.s003.docx]

| Variants | *p-value* | OR (95%CI) |
| --- | --- | --- |
| rs2802288 (G/A) | 0.005 | 1.266 (1.075-1.491) |
| rs2802290 (A/G) | 0.589 | 1.047 (0.887-1.236) |
| rs2802292 (T/G) | 0.027 | 1.207 (1.022-1.424) |
| Haplotype2 (AGGC) | 0.051 | 1.123 (0.999-1.261) |
