# Supplementary material for: Age-Based Differences in the Genetic Determinants of Glycemic Control: A Case of FOXO3 Variations
Source: PLoS One. 2015 May 20;10(5):e0126696. doi: 10.1371/journal.pone.0126696 (PMC4439071; doi:10.1371/journal.pone.0126696)
Supplement: S4 Table — TC: total cholesterol; TG: triglyceride; T2DM: type 2 diabetes; CVD: cardiovascular disease; NA: not available for missing data. a for continuous variables (TC,TG), divided by lowest, middle and highest tertile and calculated by trend chi-square test; for categorical variables (T2DM, Obesity and CVD), calculated by case-control chi-square test. b according to whether BMI ≥28 kg/m2. c self-reported CVD status. (DOCX) [file pone.0126696.s004.docx]

| *P-value* ^a^ | TC (mmol/L) | TG (mmol/L) | T2DM (%) | Obesity (%) ^b^ | CVD (%) ^c^ |
| --- | --- | --- | --- | --- | --- |
| 2802292*G |  |  |  |  |  |
| LLI | 0.132 | 0.724 | 0.505 | 0.273 | NA |
| MI_S | 0.682 | 0.779 | 0.230 | 0.598 | 0.650 |
| MI_N | 0.296 | 0.452 | 0.564 | 0.669 | NA |
|  |  |  |  |  |  |
| 2802288*A |  |  |  |  |  |
| LLI | 0.250 | 0.187 | 0.242 | 0.243 | NA |
| MI_S | 0.736 | 0.323 | 0.601 | 0.595 | 0.279 |
| MI_N | 0.624 | 0.723 | 0.475 | 0.165 | NA |
|  |  |  |  |  |  |
| Haplotype 2 |  |  |  |  |  |
| LLI | 0.341 | 0.252 | 0.439 | 0.418 | NA |
| MI_S | 0.164 | 0.389 | 0.105 | 0.792 | 0.596 |
| MI_N | 0.470 | 0.695 | 0.209 | 0.481 | NA |
