# Supplementary material for: Age-Based Differences in the Genetic Determinants of Glycemic Control: A Case of FOXO3 Variations
Source: PLoS One. 2015 May 20;10(5):e0126696. doi: 10.1371/journal.pone.0126696 (PMC4439071; doi:10.1371/journal.pone.0126696)
Supplement: S5 Table — NA: not available. (DOCX) [file pone.0126696.s005.docx]

| Variation | Title | PubMed ID |
| --- | --- | --- |
| [rs2802288](http://asia.ensembl.org/Homo_sapiens/Variation/Explore?v=rs2802288) | Association of FOXO3A variation with human longevity confirmed in German centenarians. | [19196970](http://www.ncbi.nlm.nih.gov/pubmed/19196970) |
|  | Association of the FOXO3A locus with extreme longevity in a southern Italian centenarian study. | [19415983](http://www.ncbi.nlm.nih.gov/pubmed/19415983) |
| [rs2802290](http://asia.ensembl.org/Homo_sapiens/Variation/Explore?v=rs2802290) | Association of FOXO3A variation with human longevity confirmed in German centenarians. | [19196970](http://www.ncbi.nlm.nih.gov/pubmed/19196970) |
| [rs2802292](http://asia.ensembl.org/Homo_sapiens/Variation/Explore?v=rs2802292) | Genetics and genomics of human ageing. | [21115529](http://www.ncbi.nlm.nih.gov/pubmed/21115529) |
|  | FOXO3A genotype is strongly associated with human longevity. | [18765803](http://www.ncbi.nlm.nih.gov/pubmed/18765803) |
|  | Association of FOXO3A variation with human longevity confirmed in German centenarians. | [19196970](http://www.ncbi.nlm.nih.gov/pubmed/19196970) |
|  | Replication of an association of variation in the FOXO3A gene with human longevity using both case-control and longitudinal data. | [20849522](http://www.ncbi.nlm.nih.gov/pubmed/20849522) |
|  | Genetic variations in FOXO3A are associated with Bipolar Disorder without confering vulnerability for suicidal behavior. | [21621268](http://www.ncbi.nlm.nih.gov/pubmed/21621268) |
|  | Association of the FOXO3A locus with extreme longevity in a southern Italian centenarian study. | [19415983](http://www.ncbi.nlm.nih.gov/pubmed/19415983) |
|  | The FOXO3A rs2802292 G-allele associates with improved peripheral and hepatic insulin sensitivity and increased skeletal muscle-FOXO3A mRNA expression in twins. | [20881262](http://www.ncbi.nlm.nih.gov/pubmed/20881262) |
|  | The role of genetic variants in human longevity | [20708717](http://www.ncbi.nlm.nih.gov/pubmed/20708717) |
|  | FOXO3 gene variants and human aging: coding variants may not be key players. | [22459618](http://www.ncbi.nlm.nih.gov/pubmed/22459618) |
|  | Association between FOXO3A gene polymorphisms and human longevity: a meta-analysis. | [24589462](http://www.ncbi.nlm.nih.gov/pubmed/24589462) |
|  | Shorter Men Live Longer: Association of Height with Longevity and FOXO3 Genotype in American Men of Japanese Ancestry. | [24804734](http://www.ncbi.nlm.nih.gov/pubmed/24804734) |
| [rs2764264](http://asia.ensembl.org/Homo_sapiens/Variation/Explore?v=rs2764264) | FOXO3A genotype is strongly associated with human longevity. | [18765803](http://www.ncbi.nlm.nih.gov/pubmed/18765803) |
|  | Association of FOXO3A variation with human longevity confirmed in German centenarians. | [19196970](http://www.ncbi.nlm.nih.gov/pubmed/19196970) |
|  | Replication of an association of variation in the FOXO3A gene with human longevity using both case-control and longitudinal data. | [20849522](http://www.ncbi.nlm.nih.gov/pubmed/20849522) |
|  | Association of the FOXO3A locus with extreme longevity in a southern Italian centenarian study. | [19415983](http://www.ncbi.nlm.nih.gov/pubmed/19415983) |
|  | Whole genome sequences of a male and female supercentenarian, ages greater than 114 years. | [22303384](http://www.ncbi.nlm.nih.gov/pubmed/22303384) |
|  | Analyzing age-specific genetic effects on human extreme age survival in cohort-based longitudinal studies. | [22892531](http://www.ncbi.nlm.nih.gov/pubmed/22892531) |
| rs7341233 | NA | NA |
| rs13217795 | Association of the insulin-like growth factor binding protein 3 (IGFBP-3) polymorphism with longevity in Chinese nonagenarians and centenarians. | 25553725 |
|  | Association between FOXO3A gene polymorphisms and human longevity: a meta-analysis. | 24589462 |
|  | Replication of an association of variation in the FOXO3A gene with human longevity using both case-control and longitudinal data. | 19415983 |
|  | Association of the FOXO3A locus with extreme longevity in a southern Italian centenarian study. | 19415983 |
| [rs3800231](http://asia.ensembl.org/Homo_sapiens/Variation/Explore?v=rs3800231) | Association of FOXO3A variation with human longevity confirmed in German centenarians. | [19196970](http://www.ncbi.nlm.nih.gov/pubmed/19196970) |
|  | Replication of an association of variation in the FOXO3A gene with human longevity using both case-control and longitudinal data. | [20849522](http://www.ncbi.nlm.nih.gov/pubmed/20849522) |
|  | Genetic variability of the forkhead box O3 and prostate cancer risk in the European Prospective Investigation on Cancer. | [21725602](http://www.ncbi.nlm.nih.gov/pubmed/21725602) |
|  | Low level of the mtDNA (4977) deletion in blood of exceptionally old individuals. | [20117127](http://www.ncbi.nlm.nih.gov/pubmed/20117127) |
|  | The role of genetic variants in human longevity | [20708717](http://www.ncbi.nlm.nih.gov/pubmed/20708717) |
